# Negotiating responsibility: How German healthcare professionals navigate racism between recognition and resistance

**DOI:** 10.3389/fpubh.2026.1756637

**Published:** 2026-03-17

**Authors:** Sibille Merz, Leman Bilgic, Shreyashi Bhattacharya, Elisa Mai Stüwe, Ksenia Meshkova, Rona Bird, Tuǧba Aksakal, Sharon Häussler, Yüce Yilmaz-Aslan, Patrick Brzoska, Hürrem Tezcan-Güntekin

**Affiliations:** 1 Faculty of Health and Education, Alice Salomon University of Applied Sciences, Berlin, Germany; 2 Health Services Research Unit, Faculty of Health, School of Medicine, Witten/Herdecke University, Witten, Germany; 3 Faculty of Medicine, Institute of General Practice, University Hospital Cologne, Cologne, Germany; 4 Department of Health Sciences, University of Applied Sciences Fulda, Fulda, Germany

**Keywords:** healthcare professionals, inpatient care, racialization, racism, whiteness

## Abstract

**Background:**

Research on everyday racism in healthcare remains limited, particularly in Germany. This study examines how healthcare professionals perceive their own conduct toward racialized healthcare users and explores the interpretive frameworks that shape these perceptions. By focusing on the everyday concepts that guide clinical encounters, the analysis highlights how racism is reproduced within healthcare interactions and offers insight into the knowledge frameworks and institutional processes that sustain inequities in care.

**Methods:**

We conducted problem-centred expert interviews with doctors, nurses, and members of the therapeutic professions (occupational therapists, physiotherapists and psychotherapists) working in hospitals and rehabilitation facilities in Germany. Participants were recruited systematically across three German states and interviews were conducted by telephone, in person or via videoconference. They explored respondents' observations of racism within their institutions, reflections on their own role in reproducing it, and their assessments of existing structures and further needs for effectively addressing racism in healthcare. Data were analyzed using the documentary method.

**Results:**

Twenty-one healthcare professionals were interviewed for this study. The analysis shows that implicit forms of racism remain pervasive, yet respondents displayed varying degrees of reflexivity and awareness about how racism is reproduced in everyday practice, including recognition of their own roles within these processes. Even participants engaged in antiracist efforts frequently relied on culturalizing discourses and essentialist explanatory models. While some respondents critically examined their own positionality and used their professional privilege to intervene in racist situations, such actions were typically *ad hoc* and lacked structural support.

**Discussion:**

Many respondents expressed uncertainty about how to define or analyze racism, underscoring the need for a broader public dialogue on the issue. Moreover, during their training, healthcare professionals are exposed to not only the formal curriculum but also a hidden curriculum, which subtly conveys implicit lessons about professional conduct, social hierarchies, and attitudes toward racialized patient groups. Not least, the data demonstrate the lack of institutional infrastructure to support healthcare professionals commitment to dismantle racism. Because meaningful change requires structural interventions, there is a clear need for measures that enable all healthcare professionals to contribute effectively to dismantling racism.

## 1 Introduction

Despite the pervasive acknowledgment of the ways in which racism operates within healthcare systems (1, 3, 53), empirical research on this topic remains limited—particularly within the German context where systematic data collection on issues related to race and racism has only recently begun to emerge [for example see (4–9)]. In recent years, a growing number of studies in the German context have begun to examine the experiences of racialized individuals in healthcare-related encounters, shedding light on the manifestations of racism in these settings. Tanja Gangarova et al. (51), for instance, have analyzed how Black, African and Afro-diasporic healthcare professionals as well as healthcare users self-describing as Muslim are both “othered” and silenced when seeking healthcare, contributing to a growing erosion of trust in the healthcare system. Grace Lugert-José (10) has focused on internationally recruited nurses, specifically from the Philippines [also (6)]. She demonstrates that a majority of respondents (64%) reported having experienced some form of discrimination or racism in the workplace in Germany. This is in line with findings from geriatric inpatient care, which examined the experiences of nurses of Turkish origin. Here, the majority of respondents are reported to have experienced or observed discrimination, mainly in the form of what the authors describe as xenophobic insults and rejections based on phenotypic traits and/or their actual or perceived Muslim identity (11). With regard to learning and teaching contexts, other studies have shown that medical students have very heterogeneous knowledge, awareness and perception of racism in medicine and healthcare (12). Hans Vogt et al. (13) show that racist knowledge is systematically transmitted and reproduced at different levels of German medical education (racist language in teaching materials, the under- or misrepresentation of racialized groups in seminars, racist depictions in photographs and case studies in textbooks and learning apps)—with implications for the self-image of prospective racialized doctors.

However, little is known about the role of white healthcare professionals themselves, including their attitudes toward racialized healthcare users, in reproducing racism in the German context. Elsewhere, a review on the topic (14) has found that prejudice, stereotyping, bias, and clinical uncertainty on the part of healthcare providers can perpetuate inequities in healthcare delivery. For example, Michelle van Ryn and Jane Burke (15) have found that cardiologists perceived Black patients as less intelligent, likable, friendly, and more prone to risky behavior and non-cooperation compared to white patients. Using the Implicit Association Test, Tiffani J. Johnson et al. (16) also found implicit bias toward both Black adults and Black children; the degree of bias did not vary by medical specialty, suggesting that pediatricians exhibit implicit bias toward Black children similar to physicians in other fields. Irene V. Blair et al. (17) found that physicians with stronger implicit biases consistently received lower ratings from their Black patients regarding the quality of patient-centered care compared to ratings from a reference group of white patients [also (18)]. Dimensions of experienced discrimination included a lack of time spent with providers, particularly physicians, and the feeling of not being sufficiently involved in decision-making processes.

Nevertheless, what is also crucial to examine, apart from demonstrating the existence of implicit bias and prejudice, are everyday discourses and practices by healthcare professionals, which can explain how such bias occurs and how it is reproduced within healthcare encounters. Sarah Hamed et al. (19) in a recent study in Sweden have found that what they refer to as “racialized talk”, referring to any statements or narratives that homogenize and devalue minoritized healthcare users based on their presumed race, ethnicity, culture or origin (2, 19), can reproduce racializing effects, thereby diminishing racialized individuals' access to healthcare [also (20)]. The analysis of such discourse is essential, they argue, as it is intricately tied to power relations and the maintenance—and reproduction—of structures of domination. Along similar lines, the study by Tonia Nassal and Hürrem Tezcan-Güntekin (21) has drawn on critical whiteness studies to explore white physicians' perceptions of racism and the extent to which they critically reflect on their own attitudes and practices in interactions with racialized colleagues and patients. These studies provide insight into the ways in which healthcare professionals, whether consciously or unconsciously, contribute to the perpetuation of racism by legitimizing and reproducing racialized social structures. This is particularly pertinent in contexts where overt expressions of racism are less prevalent [also (22)]. Not least, Houda Hallal (23) observes that the medical students she interviewed were personally motivated to provide every patient with high-quality medical care. Achieving this goal, however, was perceived as possible only if one critically examined one's own preconceptions and social positionality, and expanded one's professional identity to include a broader variety of perspectives.

Building on these and other studies, this article aims to contribute to the growing scholarship on racialized and racializing discourses in healthcare encounters, specifically in inpatient care (hospitals, rehabilitation facilities) in Germany. Crucially, such reproductions of racism through discourses and practices occur and are reinforced within institutional frameworks. As such, the issue here is not with specific members of staff or individual encounters but rather the institutionally entrenched and underlying forms of knowledge, patterns, and cultural assumptions that sustain these practices. Drawing on a larger study examining the forms and dynamics of racism in the German healthcare system, this article employs a diverse sample of white healthcare professionals to explore, first, how healthcare professionals perceive their professional conduct toward racialized patients, and, second, what interpretive frameworks and patterns of attribution underlie these perceptions.

In doing so, the study offers both research results and practical contributions. By focusing on how healthcare professionals explain and understand their own practices, the study helps to move forward theoretical discussions about how racism becomes built into everyday relationships and institutional routines within healthcare settings. At the same time, the empirical insights generated can guide interventions in clinical training, organizational development, and antiracism policy by identifying concrete sites where racializing practices emerge. In this way, the study not only deepens the academic understanding of racism in German healthcare institutions but also provides an evidence base for practitioners and policymakers seeking to build more equitable, reflexive, and “structurally competent” (24, 25) healthcare environments.

## 2 Methods

### 2.1 Framework

This article draws on the theoretical conceptualizations of everyday racism following Philomena Essed (26) by which she describes the often subtle but pervasive forms of racism encountered by racialized people that can manifest in individual (interpersonal, relational) and structural (engrained in government agencies, businesses and organizations at the policy and legal level) ways. Crucially, she highlights that theoretical approaches that fail to foreground the interdependence of these levels tend to place the individual outside institutional and structural rules and procedures. Instead, she argues that individual practices and forms of everyday racism are intricately tied to and maintain social and institutional hierarchies; put differently, individual practices of discrimination are embedded in and enabled by broader structural forms of exclusion. Accordingly, racism is not limited to overt, intentional acts of discrimination but constitutes a structural system of power that organizes social relations (ibid.).

The article is also grounded in perspectives from critical whiteness studies, which foreground the significance of white people in Western societies to critically engage with their own privilege and responsibility to mitigate and deconstruct racism [e.g., (27)]. Whiteness, here, refers not simply to phenotype or skin color, but to a socially constructed position of privilege, defined as a system of unearned advantages that positions socially dominant groups as normative while facilitating their access to material resources and recognition (28, 29). Accordingly, whiteness does not denote an individual identity but rather a specific social positionality, a socially and historically produced location of power within racialized (as well as gendered, classed etc.) social orders. Moreover, the assumption is that from this position of privilege, racism frequently remains imperceptible; as a result, white individuals often reproduce it, intentionally or not, and continue to derive benefits from it (29). White privilege, then, is not about individual acts of malice but about “invisible systems conferring dominance” [(29), n.p.] and complicity in the reproduction of racialized orders. The responsibility to engage with and address (their own) racism is thus placed on white individuals; as Khan (30) argues, white silence in the face of racism functions as a core strategy in reproducing racism.

### 2.2 Sampling

A sample of around 20 participants was deemed appropriate to reconstruct the attitudes and practices of healthcare workers both in depth and breadth. Each case was to be analyzed in depth for its unique contribution to uncovering and reconstructing underlying structures and frameworks. The sampling strategy ensured representation from three distinct professional categories—physicians, members of the therapeutic professions (occupational therapists, physiotherapists and psychotherapists), and nurses—with seven participants in each group. Within these groups, efforts were made to include at least two individuals working specifically in the rehabilitation field. Additional selection criteria accounted for diversity in geographic settings (urban vs. rural) and Germany's socio-historical context, particularly the legacy of the division between East and West Germany, following the principle of purposive, specifically maximum variation sampling (31). To reflect these factors, data collection was conducted across three federal states: Berlin (an urban area encompassing both formerly East and West Germany), Baden-Württemberg with a focus on the rural Schwäbische Alb region (formerly West Germany), and Thuringia (offering a mix of urban and rural settings, formerly East Germany). To mitigate low response rates within the rehabilitation sector, we leveraged professional networks established through prior research to directly contact relevant institutions, which resulted in the inclusion of a limited number of participants from outside the three target states, i.e., from North Rhine-Westphalia where these networks were concentrated.

Sampling also sought to capture a range of levels of professional experience. Prior research indicates that while more seasoned professionals may draw from a wider array of clinical encounters, younger adults often demonstrate greater awareness of racial dynamics (32). Although this variable did not directly shape the initial sampling process, it was integrated into the analytical process to inform possible refinements in subsequent participant recruitment. Focus of the sampling strategy were white individuals; however, in our invitation to participate in the study we asked people who self-identified as “not negatively affected by racism” to participate: We avoided the term “white” because in common perception in Germany, especially among those outside scholarship on racism—“white” is often understood narrowly as referring to skin color rather than as a social position associated with structural privilege.

Ethics was obtained by Fulda University of Applied Sciences, Az.: EK230327-AuEnd.

### 2.3 Recruitment

Recruitment was conducted systematically and in alignment with the defined sampling strategy. Contact information for all hospitals and rehabilitation centers within the three selected German states was systematically compiled. Where possible, personalized invitations were sent directly to medical and nursing directors via e-mail, inviting their participation and requesting that they share the study details with relevant staff. In instances where direct contact details were unavailable, general inquiry forms on institutional websites were used to establish communication. To counterbalance the initial predominance of hospital-based respondents, additional recruitment efforts focused on engaging professionals from rehabilitation facilities, drawing on existing professional networks to facilitate access.

Individuals who expressed interest in participating in the study contacted the research team via e-mail and interviews were subsequently scheduled.

### 2.4 Data collection

Interviews followed the precepts of problem-centered expert interviewing (33). The research team developed a topic guide through multiple rounds of feedback from other team members and the project advisory board. The guide addressed themes including respondents' understanding and perception of racism, their personal strategies for managing racism, and interactions with racialized patients. Additional topics included self-reflexivity regarding respondents' own privileged positions, current approaches to managing racism within their institutions, and perceived needs and preferences for future interventions (see Table 1). Interviews were conducted in person where possible, over the phone or via digital platforms such as Zoom or Webex. In-person interviews were conducted at a place chosen by participants, usually at their workplace. No one else besides the participant and the researcher was present during the interview. All but three interviews were conducted by SM while HTG conducted three interviews with participants in central leadership positions to professionally match interviewer and interviewee more closely [for a critical discussion of the insider/outsider positionality see e.g., (34)]. All participants gave their written consent to be interviewed and for their data to be used.

**Table 1:** Topic guide.

**No**.	**Topic**	**Question(s)**	**Possible follow-up questions**
1	General opening: facilitating the start of the conversation, stimulating narration	•Could you describe the position in which you work and how long you have been in this profession?•What motivated you to take part in our interview study?	
2	Attitudes toward racism: how racism is understood, perceived, and addressed	•Have you witnessed situations in which patients or colleagues have experienced racism in your institution?	Can you tell me of specific situations to illustrate this? In which area of your institution do you witness this? Why do you think this particular person was racially insulted or mistreated? Were other people present and if so, how did they respond? What happened after this? Are there specific phases of the hospital stay during which racism occurs more frequently? Have you witnessed that patients who have experienced racism in your institution interrupt their treatment or self-discharge early? If so, how do you deal with such cases?
3	Construction and interpretations of diversity	•Do you perceive differences between patients based on their origin, ethnicity, religion, or culture, for example regarding how they experience illness? •Are patients' origin, religion, or culture taken into account when planning bed assignments, room allocation, or therapy decisions? If so, in what way?•I would like to describe a hypothetical situation and ask you to discuss it: *Imagine an older, female Turkish-born patient on your ward who complains of severe, persistent pain with an unclear diagnosis. The patient asks for a higher dose of her pain medication, but your colleague advises against increasing the dose. She comments: “They always exaggerate anyway.”* Have you encountered such situations or similar ones? How would you respond? If this colleague was your superior, how would you respond?	If so, do you have any idea how these differences could be explained? What does that mean for your everyday practice? In your view, should differences be made in treatment? What role do other characteristics (for example gender, age, or disability) play in these decisions? Could you give me an example?
4	Critical self-reflection	•Have you ever noticed that you yourself hold prejudices against people based on their origin, religion, ethnicity, or culture?•To what extent do these characteristics influence your professional relationship with your patients?	Can you give me an example? Would you also describe that as racist? How would you define racism in general? How did you deal with it? What do you think about it today? Do you notice similar thoughts in other areas of your life? People who do not commonly experience racism themselves are often described as privileged. How would you assess your own privilege? Are there areas in which you feel more or less privileged? Have you ever considered how your privilege might influence your behavior or interactions with patients? How might your privilege affect your perception of racism?
5	Existing infrastructure and strategies from the professionals' perspective	•How are experiences of racism addressed in your institution?•Are there specific contact persons for discrimination? If so, how do those affected find out about these resources?•What would you wish for regarding how experiences of racism are handled in your institution?	How are patients with limited German language skills treated in your facility? Do you have any specific suggestions for areas where improvements could be made?
6	Conclusion, room for own suggestions	•Is there anything else we have not addressed yet?	

Interviews lasted between 25 and 80 min. They were audio-recorded and transcribed verbatim. The software Sasaya (35) was used for automated transcription and each transcript was checked manually for errors and anonymization.

### 2.5 Data analysis

Data analysis followed the documentary method as developed by Ralf Bohnsack et al. (36). This method aims to reconstruct the practical experiences of individuals and groups in specific milieus or organizations, providing insight into their orientations for action that emerge in practice. The method is characterized by a shift in the analytical focus from *what* is said to *how* it is said, and the specific orientation and habitus of the interviewed person manifesting therein. It ultimately aims to construct, through the repeated and iterative comparison of different segments from different interviews or within the same interview, different abstract types, what Bohnsack refers to as “sense-genetic types” [on the tension between critical research and the documentary method, see (37)]. Crucially, these types do not represent groups of respondents but rather clusters of specific orientations or attitudes toward a specific problem or question and the resulting practices. Accordingly, the types are not absolute or mutually exclusive; instead, a single respondent may align with multiple types at once. Moreover, we emphasize that, following Pierre Bourdieu (38), attitude and action are deeply intertwined within social practices—attitudes are part of the tacit knowledge structuring social practices. Put differently, we do not distinguish between attitude and practice but view attitude, as an internalized pattern of perception, thought and action, as enabling or constraining an individual's action. While habitus does not *determine* action, it ensures that the individual is more or less inclined to act in a certain way.

Adopting Bohnsack's conceptual frame but simplifying it given our sample size and aiming to analyze data collectively from multiple perspectives (some of us have directly experienced racism while others have not), our data analysis followed three analytical steps. First, each member of the core research team (LB, RB, SB, SH, EMS, SM) was assigned a specific number of interviews and extracted all text segments pertaining either to an interviewees' perceptions of their professional conduct toward racialized patients, or their actions in relation to these patients. These segments were sorted into main themes (perception/practice) and subthemes and logged in MS Excel. Second, the extracts were jointly analyzed in weekly sessions for “reflective interpretation” which entailed a semantic and comparative analysis. This involved scrutinizing not only what was said but also how it was expressed, while comparing related statements within a single interview and across different interviews. The analysis emphasized understanding the respondent's perspective without assessing the truthfulness of their statements, focusing instead on what these statements indicate about their attitudes or viewpoints. Last, one researcher (SM) developed a typology of attitudes and practices based on these interpretations, which was regularly discussed and refined in the larger research team. The move from individual cases involved constructing each type by synthesizing statements from multiple interviews or cases.

Our reconstruction of specific types is not merely an intellectual exercise but serves as an entry point to identify regular and habituated ways in which racism is reproduced in everyday practice to stimulate discussion on how to eventually dismantle racism in healthcare delivery. While interventions at the individual level, for example through antiracist education and diversity training, have been proven to be often ineffective (39), deconstructing these patterns can open up new possibilities for developing more effective interventions against racism at the institutional level. This deconstruction entails scrutinizing the historically evolved, culturally embedded, and professionally internalized bodies of knowledge that continue to inform institutional norms and everyday interactions within medicine and healthcare.

### 2.6 Research team and reflexivity

The team consisted of diverse positionalities vis-à-vis and experiences of racism (three researchers identify as white while all others have experienced racism based on different phenotypical or cultural markers), levels of experience and professional status (including students as well as research team leaders and professors). Different disciplinary backgrounds, spanning medicine, epidemiology, public health, sociology, psychology, education and anthropology, shaped our approaches to data collection and analysis. The team's multi-perspectival composition and different social positionalities thereby enriched the analytical process; drawing on our diverse standpoints and experiences allowed for a more nuanced interpretation of the material and helped surface insights that might otherwise have been overlooked. At the same time, this collaborative approach countered academia's often-divisive logic as it fostered solidarity and mutual care within the team. In practice, this meant that potentially harmful material could be allocated in a way that protected specifically racialized colleagues from the risk of re-traumatization. For example, interviews that were known to feature explicitly racist content were analyzed by a researcher self-identifying as white. This way, the team safeguarded the wellbeing of colleagues while maintaining analytical rigor. Moreover, this diverse composition also required an explicit recognition of the unequal distribution of experiential knowledge. Colleagues of color held the authoritative voice when interpreting data that touched on racialized experiences and racializing practices, ensuring that analyses were grounded in lived realities rather than only theoretical assumptions. This approach not only strengthened the credibility and nuance of our interpretations but also positioned reflexivity as a central and ongoing ethical practice.

## 3 Findings

### 3.1 Sample description

A sample of 24 respondents was recruited, out of which 21 respondents were selected for this article, consistent with the methodological requirement for a relatively small but information-rich dataset (see Table 2). Of those 21 respondents, nine were doctors, eight were nurses and four were members of the therapeutic professions (one physiotherapist, two occupational therapists and one psychotherapist). The majority of respondents worked in a hospital setting while four worked in a rehabilitation facility. Twelve respondents were based in Berlin, four in the southern state of Baden-Wuerttemberg, three in Thuringia and two in North-Rhine Westphalia. Eleven respondents identified as female while 10 identified as male. Ten respondents occupied managerial positions in their institutions.

**Table 2:** Sample description.

**No**.	**Study ID**	**Gender**	**Age group**	**Professional experience (years)**	**State (B/BW/NRW/TH)^a^**	**Profession**	**Institution (H/R)^b^**	**Size of institution^c^**
1	0301	F	45–64	20	B	Medicine	H	600
2	0302	F	45–64	46	B	Nursing	R	60
3	0303	M	45–64	25	BW	Nursing	H	2,400
4	0304	M	45–64	20	B	Medicine	H	800
5	0306	F	45–64	41	BW	Nursing	H	350
6	0307	F	45–64	40	B	Nursing	H	900
7	0309	F	45–64	38	B	Nursing	H	260
8	0311	F	45–64	21	BW	Therapeutic professions	R	120
9	0312	M	45–64	22	B	Medicine	H	450
10	0314	F	45–64	30	BW	Therapeutic professions	H	20
11	0317	M	45–64	30	TH	Medicine	H	1.450
12	0318	M	30–44	15	TH	Therapeutic professions	H	900
13	0320	F	45–64	39	B	Nursing	H	150
14	0321	M	45–64	15	TH	Therapeutic professions	H	640
15	0322	M	45–64	30	B	Medicine	H	1.000
16	0324	M	30–44	14	B	Medicine	H	1.000
17	0325	M	30–44	20	B	Nursing	H	600
18	0326	M	30–44	14	B	Medicine	H	370
19	0327	F	30–44	17	B	Nursing	H	1.000
20	0329	F	30–44	13	NRW	Medicine	R	1.000
21	0330	F	45–64	40	NRW	Medicine	R	300

^a^B, Berlin; BW, Baden-Wuerttemberg; NRW, North Rhine-Westphalia; TH, Thuringia.

^b^H, Hospital; R, Rehabilitation facility.

^c^No. of beds, rounded.

### 3.2 Typology of attitudes and practices

Most respondents demonstrated a pronounced interest in the topic and explicitly recognized its relevance, even if the intensity of this recognition varied across cases. Moreover, the majority displayed at least some degree of self-reflection, indicating an awareness of their own social positionality in relation to racialized power dynamics. However, they did vary significantly according to the kinds of interpretive frameworks and patterns of attribution that underlay their perceptions of, and practice with, racialized healthcare users and colleagues. As such, we constructed five different types of orientations or attitudes (for an overview see Table 3); to reiterate, these do not necessarily designate discrete (groups of) respondents but rather clusters of attitudes toward a specific problem, and forms of action resulting from them. While individual interviews occasionally contained internally inconsistent or divergent statements, the quotations presented here were selected to illustrate the core characteristics of the analytic types rather than to exhaustively represent the full range of views expressed by any single respondent.

**Table 3:** Typology.

**Type**	**Central characteristics**
Critical reflexivity	Recognizes racism and can define it critically in terms of power and structural embedding; is able to position oneself within societal power structures and identify personal privilege, especially whiteness; addresses personal responsibility in the reproduction of racism; applies an intersectional approach; uses its own institutional power to counteract racism.
Antiracism	Prioritizes active intervention against racism over self-reflexivity; anti-racism is part of its own identity as engaged citizen; often focuses on extreme right-wing positions, especially the *Alternative für Deutschland* (AfD) party; while this type recognizes its privilege, reflexivity is subordinated to active intervention.
Liberalism	Is sensitive to racism but has a limited understanding of racism as intolerance; implicitly refers to concepts such as multiculturalism, or color blindness; has a humanitarian understanding of anti-racism as partnership; questions of privilege tend to be denied or answered in a limited way, whiteness is not addressed; nonetheless, uses its own position to actively combat racism.
Foregrounding intentionality	Views as racist only those who act accordingly; focuses on individual expressions of racism; legitimizes racist incidents as thoughtlessness or reflex; has limited awareness of structural racism, including its own internalized racism; denies its own privilege.
Skeptical dismissal	Does not recognize racism as a structuring principle of contemporary societies; believes racism is a problem of the past; reverses perpetrator-victim roles; does not reflect on own privileges and power, takes defensive approach; questions legitimacy of the research endeavor.

#### 3.2.1 Critical reflexivity

What we describe as critical reflexivity is characterized by the recognition of racism including its structural dimensions and historical legacies. Respondents exhibiting this cluster of attitudes and approaches to practice were able to define racism confidently and as structurally embedded. For instance, a gynecologist and medical director from Berlin argues:

*Racism is a form of discrimination based on origin or difference, and ultimately, you could perhaps summarize it as applying to everything that is not… Well, we live in a very patriarchal society, and anything that does not conform to the male, white mainstream is initially different and marginalized* (0301, female, medical director, Berlin)

Similarly, a junior doctor based in Berlin frames it as such:

*But as far as this structural racism is concerned, which I actually find so, what's the word, interesting is a difficult word, but I find it exciting because for a long time I simply wasn't aware of how deeply rooted it is and how often, as an outsider, you actually actively reproduce it without realizing it* (0324, male, resident, Berlin).

Moreover, critical reflexivity includes the ability to analyze one's own position in societal power structures and name one's own privileges, specifically whiteness. The junior doctor cited above notes:

*If racism is the concept that, above all, a white population group applies negative standards to other population groups, then I must describe myself as privileged, because I naturally belong to the group that tends to oppress others and is on the oppressor side* (0324, male, resident, Berlin).

Critical reflexivity also addresses personal responsibility in reproducing racism on a day-to-day basis. The senior doctor notes:

*So we're all racist, that's just how it is. We're all sexist, we're all racist. And it's about recognizing that or trying to be less racist, so to speak*. (0301, female, medical director, Berlin)

The junior doctor adds:

*What this privilege entails is that at some point you realize that if you don't engage with the issue, you are somehow always a supporter of it. That's what's so problematic, I think. If you don't raise awareness, it keeps getting reproduced and thus perpetuates the system and also causes people suffering, so to speak. So, yes, I am privileged, but then I also have to share the responsibility for it*. (0324, male, resident, Berlin)

Here, the respondents acknowledge their own complicity in the routine reproduction of racist structures. They recognize their privilege and responsibility in the ongoing, ordinary enactment of racialized dynamics. However, what becomes apparent in the first respondent's statement is also a rather swift diversion to the theme of sexism. This indicates that, even for critically engaged individuals, sexism appears easier to articulate, or has been a more firmly established topic within German discourse on inequity.

Moreover, critical reflexivity involves the ability to analyze power relations through an intersectional lens:

*Now we have a situation that happens every day. There are two aspects to this: violence in obstetrics and racism, so to speak. In this case, these two problems are intertwined*… (0301, female, medical director, Berlin)

*I can say briefly that, as my ex-girlfriend liked to say, I am a white cis man, I come from northern Germany, and I was relatively unaffected by the issue* (0324, male, resident, Berlin)

While the first quote demonstrates that the physician analyzes occurrences on her clinical ward through the lenses of intersecting systems of oppression, the resident describes his own location within intersecting power structures, naming racism as well as sexism, cis normativity and region as shaping his position of privilege.

Crucially, the typology elaborated here is not limited to attitudes, but addresses concomitant orientations toward practice. Critical reflexivity thus translates into concrete action and intervention, with respondents mobilizing their own privileged positions in their respective institutions to dismantle racism. For example:

*In that case, I held a racism workshop, in quotation marks, with my senior midwife and one of my senior physicians. I talked about racism, so to speak, and simply put the examples that had happened in recent weeks on a PowerPoint slide* (0301, female, medical director, Berlin)

*Yes, I have to say, I did that in [town in which he studied] back then, too, I already made it an issue, and there, too, I already took a stance and said that I don't feel that way*… (0324, male, resident, Berlin)

While both respondents seek to responsibly use their privilege to fuel change in their respective organizations, they do so in single, *ad hoc* rather than systemic interventions. Occurring at different positions of power, they reflect an awareness of power dynamics and individual responsibility, but their interventions remain limited in scope nonetheless, lacking the institutional infrastructure necessary to produce sustainable change. While this may not be surprising for the case of a junior physician, the lack of resources for the medical director to counter racism on her ward demonstrates the absence of institutionally anchored structures to ensure racism is addressed effectively. Such isolated events may raise awareness or address immediate concerns but are insufficient to tackle the entrenched, structural determinants of health inequities, specifically racism.

However, this type is not without its contradictions and ambivalence. On the one hand, respondents whose perceptions align with the critical-reflexive type sometimes exhibited a classist tone, strongly reflecting middle- to upper-class norms, values and modes of speaking. This sometimes even extended to statements that attributed the largest share of responsibility for institutional racism to nurses while simultaneously positioning themselves as more reflexive, justified by their own university education. This simplistic equation of self-reflexivity with educational and professional status highlights a clear contradiction in their accounts. On the other hand, the porosity of their argumentation becomes visible when one respondent makes a fleeting reference to “real racists” (0301, female, medical director, Berlin) (presumably in contradiction to “unreal racists”), aligning her more with what we describe as the cluster “foregrounding intentionality” below. Overall, however, clear articulations of the mechanisms of structural racism and its routine reproduction through social practices dominate in this cluster.

In sum, critical reflexivity combines strong self-reflection with active intervention.

#### 3.2.2 Antiracism

The second cluster of attitudes and orientations to action, which we have termed antiracism, also involves regularly combating racism within and outside institutions. In fact, the active intervention against racism is the primary focus and driver of the orientation toward racism associated with this type, and part of respondents' self-identification as politically engaged citizens. In particular, respondents aligning with this type are often engaged in movement-building against the German far-right party *Alternative für Deutschland* (Alternative for Germany, AfD), or perceive antiracist activism as part of their overall political commitment, frequently from within a labor union. However, in contrast to the critically reflexive type described above, the attitude and practical orientation of antiracism does not involve in-depth reflections of social positions and their attendant privileges. While respondents aligning with this type are aware of their privileged position in socio-economic terms, specifically vis-à-vis global divisions of labor, they do not frequently engage with their own whiteness and subordinate this reflection in importance to active intervention. We will exemplify this orientation through original quotes from our data.

First, the antiracist type focuses primarily on active intervention against racism:

*And, um, it's really important to me that no, um, racism happens there, and I also try to do something against it… So (..) [I'd like to know] like, how severe is the extent and so on. And what possibilities are there as well*. (0307, female, nurse, Berlin)

*Well, my attitude is also generally known, so in my presence they really rarely make such [racist] remarks*. (0307, female, nurse, Berlin)

*I know that even within my leadership team, the topics are often, um, also exhausting, because I keep bringing them up… I also signed the Diversity Charta for our organization years ago. I did this very deliberately… because I wanted to work on this issue. And that sets me apart from, um, the managing director. He has nothing against it, but, um, for me there was a lack of active – yes, active measures that could be taken* (0320, female, nurse, Berlin)

This activism is part of respondents' overall self-identification as politically engaged citizens:

*Um, well, because this is an important issue for me, you know, because I am a politically minded person. And, um, well, I have already witnessed a lot of racism, or, yes, racism directed against patients, in my almost twenty years in the hospital environment* (0325, male, nurse, Berlin)

*Well, I cannot keep my mouth shut when it comes to this [racism]. This is, well, as I said, I am very active in the union, we have also been active in industrial action, organized strikes* (0307, female, nurse, Berlin)

As part of this commitment to democracy and justice, respondents aligning with this type are particularly active in the fight against the AfD party, the first mainstream right-wing party in Germany in federal government since World War II, which currently ranks second in the polls behind the leading Christian Democrats (CDU). This respondent notes:

*I am also very active in the union, and we are currently organizing in the direction of – well, first, as a reaction to the Correctiv-Enthüllung [news story by the collective Correctiv that demonstrated how AfD politicians and other right-wing activists had met to develop strategies to “remigrate” racialized people in Germany] but also we had been thinking earlier that we want to do something about the European elections, against the AfD* (0307, female, nurse, Berlin).

*And only last week I participated in a seminar about right-wing populism in the everyday and the next day, as part of our managerial meeting, I told my colleagues about it. And it made me very sad that nobody asked any questions about it*. (0320, female, nurse, Berlin)

In this framing, racism is closely associated with, and at times reduced to, the politics of far-right extremism. This equation risks obscuring the ways in which racism is reproduced within centrist or even progressive institutions, and instead positions racism as a pathology located solely at the political margins. Consequently, struggles against racism are frequently articulated through opposition to right-wing populism, rather than through a broader interrogation of structural and everyday forms of racial inequity.

In contrast to critical reflexivity, attitudes and practices of the antiracist type do not center on reflecting upon privileges vis-à-vis racism, or the responsibility that ensues from this reflection. Instead, respondents aligning with this type either deflect by turning to forms of socio-economic (de)privilege, for which they are very much sensitized, or downplay the focus on privilege itself by focusing on the coincidental nature of birth and citizenship:

*No, I am privileged…. I come from a middle-class, German family. I am an academic's child, eventually I'll certainly inherit something. So, I am very much ahead of a lot of people*. (0325, male, nurse, Berlin)

*Of course, I can see that I am privileged, right? To lead the kind of life I am leading… But I know that we all here live at the expense of others, the way we live. But what is the consequence of this? Is this because I don't experience racism or simply because I was born here and live here? You know what I mean?* (0307, female, nurse, Berlin)

*Well, it's not that I have not experienced discrimination. There is a topic where I am very much deprivileged. Because I do not have children. But I am very much privileged. I don't really get questioned much. Sometimes you could call it the whole “East–West thing.” I was born in the East, but I've got a pretty broad range of experiences. When people ask if I'm from the West, I usually say, ‘No, I'm from the North'… But yeah, apart from that, I'm really quite privileged. And, um, you know, being questioned—like how, what, where—that's not really comparable to what other people might go through, or actually do go through. Even those questions like, ‘Where are you from?' or ‘Where are you* ^*^really^*^ *from?'—those aren't the kinds of questions I constantly face as a woman who grew up in Germany, lives here, and has always been her*e (0320, female, nurse, Berlin).

The first two quotes demonstrate that while respondents are aware of their own privilege, they mostly attribute it to notions of social class or their citizenship and upbringing in Germany rather than the intersections of class, nationality and whiteness. The use of the collective “we” in the second quote (“we all here live at the expense of others”) further homogenizes a collective Germanness as a *white* Germanness, invisibilizing Germans of color. In the final quote, the respondent both acknowledges and deflects questions of privilege. She initially identifies her lack of children as a form of disadvantage and, despite beginning by admitting her privileged position, subtly reveals that she conceals her upbringing in the GDR because she perceives it as a potential reason for marginalization. While she subsequently reiterates her privilege, she frames it primarily in relation to her gender and national identity. Importantly, she nonetheless demonstrates awareness of the subtle forms of racism that people of color in Germany encounter, particularly through exclusionary questions such as “Where are you really from?” Yet, she avoids confronting her own whiteness as the key reason she is spared these experiences, leaving the structural dimension of her privilege unexamined. Despite this type's much-needed focus on intervention, this blind spot risks reproducing racism as it does not decenter and deconstruct privileged positions.

In sum, the emphasis here lies on practical action, without a thorough critical examination of one's positionality and privilege *qua* whiteness.

#### 3.2.3 Liberalism

Similar to the previous type of attitudes and orientations to practice, liberalism rests on an acknowledgment of the relevance of racism and respondents use their own powerful positions to seek to dismantle it. In contrast to previous clusters, however, racism is equated with intolerance, drawing on liberal-humanist discourses around multiculturalism, which focus on coexistence and inclusion while often leaving the structural roots of persistent inequity unexamined:

*Well, I would say it has something to do with intolerance. The moment I behave intolerantly towards my counterpart in the sense that I judge all his inner attitudes or his appearance or his religion or whatever as wrong and negative*. (0303, male, nursing director, Baden-Wuerttemberg)

*I think this is a very, very important issue and one that needs to be handled sensitively. Whether it's because of radicalism or right-wing or left-wing extremism, or simply strong opinions, um, and sometimes you have to promote tolerance on a daily basis*. (0321, male, occupational therapist, Thuringia)

Such discourses of multiculturalism often present themselves as celebratory and inclusive:

*Among my colleagues, we have become quite a colorfully mixed group, I would say. We have a colleague who comes from South Korea. We have two Syrian colleagues. We have a Palestinian*… (0317, male, medical director, Thuringia).

*Here in [major German city], we have a colorful diversity, and of course we have specific topics at our hospital accordingly* (0303, male, nursing director, Baden-Wuerttemberg).

Such narratives tend to reduce complex social inequalities to a matter of cultural or aesthetic representation, depoliticizing racialized hierarchies and rendering whiteness invisible. Moreover, they risk reinforcing stereotypes by essentializing racialized groups as repositories of a specific culture, while positioning inclusion as contingent on performative recognition of these differences rather than the redistribution of power and resources.

Because the pervasiveness of structural racism and its structuring of social relations is absent within this cluster of attitudes and practices, a humanitarian understanding of anti-discrimination that centers “partnership” prevails:

…*on the interaction level, on the social level, on the partnership level, it really has to be based on partnership. There's no hierarchy there*… (0312, male, attending physician, Berlin)

The assumption here is that social interactions can occur outside of societal hierarchies, with the respondent positioning himself (a white man and senior physician) as neutral and unmarked. This renders privilege invisible and reproduces inequality under the guise of impartiality.

Because of this lack of sustained engagement with structural inequity, the question of privilege vis-à-vis racism is denied or rejected:

*I don't know if I would say I'm privileged if something like that hadn't happened to me. I'm just in the wrong country, or fortunately in the right one. So, as the person I am, with my values, culture and religion, I would probably also be a victim of racism in another country*. (0303, male, nursing director, Baden-Wuerttemberg)

*Well, not really. Because I have an addiction problem and have experienced other prejudices, or still do, so I have some idea of how it feels to be marginalized in a racist way*. (0302, female, nurse, Berlin)

The first statement emphasizes the perceived happenstance of birth, and advances the assumption that white people can equally experience racism, emptying racism of its historical anchoring in colonial expansionism and imperialist domination. The second quote deflects attention from the respondent's own racial privilege, equating the experience of exclusion due to her substance addiction with the systemic oppression of racialized groups. In both cases, the operation of whiteness as a position of unmarked advantage is obscured, collapsing structural forms of racial inequity into coincidence or individual experience.

Nonetheless, this type tends to actively use their position to advance anti-discrimination targets in the institution or intervenes when observing instances of racism:

*But I have a colleague who occasionally makes comments about patients like that. And actually, when that happened in my presence, once, I immediately made it clear that it was not acceptable* (0317, male, medical director, Thuringia)

*Yes, yes, I've always said something, yes* (0302, female, nurse, Berlin)

*Now, as a supervisor at the hospital, if I encountered something like that in my daily work, I would of course say something* (0312, male, attending physician, Berlin)

…*And the four of us constitute the hospital management and we are firmly convinced that we must do everything in our power to counteract racism* (0303, male, nursing director, Baden-Wuerttemberg)

Here, we can witness differing degrees of commitment and approaches to antiracist intervention. These range from vague expressions of intent—such as “saying something” when witnessing discrimination, though often without clarity regarding what such an intervention would entail—to hypothetical statements like “I would say something,” which suggest a lack of concrete experience or prior engagement in such situations. At the other end of the spectrum are those who have made antiracism or diversity work a central aspect of their professional practice. Overall, however, most participants described forms of engagement that were primarily reactive: they tended to intervene only when explicit incidents of racism occurred, rather than engaging in proactive or structural forms of antiracist action.

The liberal cluster of attitudes and approaches to practice involves an acknowledgment of racism but primarily interprets it through a cultural-humanistic lens rather than a structural one.

#### 3.2.4 Foregrounding intentionality

Another cluster of attitudes we constructed from our data can be described as a foregrounding of intentionality. Unlike the previously discussed clusters, this type does not conceive of racism as a structuring modality that shapes social relations, modes of thought and everyday practices. Rather, racism is acknowledged only in cases of deliberate, intentional acts. Put differently, although attitudes represented by this type involve recognizing racism as morally wrong, the label “racist” is restricted to those who act in consciously discriminatory ways. In contrast, internalized stereotypes and racialized constructions are perceived as natural or self-evident; what matters, in this view, is whether one acts upon these assumptions. As a result, attitudes and practices of this type demonstrate little awareness of structural racism and rest on a failure of individuals to recognize their own role in its reproduction. Accordingly, questions of privilege remain unaddressed, often denied or outright rejected. At the same time, respondents aligning with this type may actively intervene when confronted with overt instances of racism; however, due to their narrow definition, they frequently overlook more subtle and pervasive forms of racialization. We will go through these dimensions one by one to provide evidence for our argument.

First, respondents whose attitudes we categorized as intentional only recognize racism in cases of deliberate acts. Consider the following excerpts:

*I don't believe I am racist. I'd rather not, but I, I, well, in order not to be, I think about it. And I would say that I am probably quite consciously friendly toward people* (0329, female, specialist physician, North Rhine-Westphalia)

*Well, the thing is, we've only experienced this kind of everyday racism from patients once. I think it's more likely that the patient had a delusion. And that was then amplified, he tended to reject the employees, the international ones. And [otherwise] um, it's mostly thoughtlessness which is actually already racist* (0309, female, nurse, Berlin)

The first quote demonstrates that the respondent does not perceive herself as racist, grounding this assumption in her reflexivity and the claim that she is therefore friendly toward racialized patients and colleagues. Implicit in this reasoning is the notion that personal friendliness serves as sufficient evidence of being “non-racist.” The second excerpt, in turn, not only highlights the respondent's pathologization of a patient who expressed overtly racist views, but also exemplifies a tendency to trivialize racism as harmless or unintentional. This occurs even while the respondent simultaneously acknowledges that such behavior could indeed be described as racist.

In this framing, the presence of internalized stereotypes and racialized constructions are not denied but perceived as natural, or even reflexive. This is exemplified by the following quotes:

*I: Would you describe this reflex you described as racist? B: I'd rather not, no. I don't believe I am racist. I'd rather not*… (0329, female, specialist physician, North Rhine-Westphalia)

*So, with discrimination and racist remarks or whatever, it's always a form of aggression and, in my opinion, it's often suppressed and people often say that it shouldn't exist, but I personally think that's nonsense. I think every person has this aggression inside them, and it's important to be aware of it and not let it turn into action* (0318, male, psychotherapist, Thuringia)

Such framings of racism as a reflex reflect a depoliticized understanding. In this view, racism is construed not as a structural or systemic condition but as an involuntary, almost automatic biological reaction—something that “just happens” at the level of perception or habit. This interpretation allows individuals to acknowledge the existence of racialized responses while simultaneously displacing responsibility for them. By attributing racism to reflex, it is rendered a matter of instinct or social conditioning rather than of power, ideology, or position within racial hierarchies. Such a framing also functions defensively: it maintains an image of the self as reflective and well-intentioned, even while reproducing racialized assumptions. Racism becomes something that one can notice or observe in oneself without it threatening one's moral integrity or professional identity as one does not act on it. Ultimately, conceptualizing racism as reflex collapses the distinction between awareness and accountability: one can be aware of racism without necessarily challenging the conditions that sustain it.

Accordingly, respondents in this category have little or no awareness of structural racism but frame it as an individual attack, insults or acts of ignorance:

*Well, attacking someone because of their origin, um, verbally, that can also be through ignoring them, ignoring someone permanently. That's also a form of exclusion* (0309, female, nurse, Berlin)

*I: Are there situations where you realize that you are perhaps not completely free of it and have somehow fallen back into such a cliché? B: Well, I don't think so in terms of concrete actions. I think I certainly already have a perception reflex* (0329, female, specialist physician, North Rhine-Westphalia)

Here, too, rather than understanding racism as a system of power that organizes social relations and material resources, the respondents interpret it primarily through the lens of individual behavior. This individualized framing not only obscures the embeddedness of racism in broader social, political and institutional structures but also limits the respondents' capacity to recognize their own participation in its reproduction. Consequently, racism becomes a matter of personal morality or interpersonal civility, detached from the structural conditions that sustain racial inequity.

Indeed, respondents whose attitudes fall in this type do not, or not fully, acknowledge their own privilege in racialized hierarchies. When prompted about their privilege, they replied:

*No, not at all [laughs]. No [laughs]. So I don't see myself as privileged in any way. I see it more as an upbringing… And I do see that we in Germany are relatively well off and that many of us as a population are simply privileged, all of us, so to speak, yes*. (0309, female, nurse, Berlin)

*Of course, I don't experience racism in Germany. In that respect, I am privileged, for sure. […] From my own professional experience, I recognize discrimination based on my gender* (0329, female, specialist physician, North Rhine-Westphalia)

Similar to statements associated with previous types, these quotes exemplify that respondents do not make their whiteness explicit and find it difficult to speak about racism. They also deflect and turn to those parts of their identity that are marginalized, for example due to sexism. Another respondent did recognize his own privilege but failed to link it to everyday racist structures or address his own whiteness, rather foregrounding his upbringing in the German Democratic Republic as a grandchild of Nazis and thus focusing only on overt forms of (state) racism:

*Yes, I'm full of it [privilege]. So, um, I was socialized in the GDR, until I was ten or nine years old, both my grandfathers, served in World War II… I have heard terrible things, um, about Black people, about Jews… so that's part of my socialization* (0318, male, psychotherapist, Thuringia)

In sum, then, similar to other respondents, respondents whose attitudes fall into this category demonstrate limited reflexivity regarding their own privileged positionality and their complicity in reproducing racialized structures in everyday life.

The distinguishing feature of this type is that it reduces racism to individual intention while systematically minimizing its structural dimensions.

#### 3.2.5 Skeptical dismissal

Lastly, some respondents articulated either skeptical or even dismissive attitudes and orientations toward the issue at hand. They not only failed to acknowledge the importance of examining racism within healthcare and broader social contexts, but also rejected the very notion of racism as a persistent social problem. Their narratives frequently involved the inversion of perpetrator-victim roles, thereby deflecting attention from structural inequities and precluding any meaningful engagement with their own privilege and positional power. In sum, attitudes and practices of this type actively deny the existence or severity of racism and demonstrate a limited capacity to critically reflect on racial inequity. We have therefore opted to describe attitudes and practices of this type as skeptical dismissal.

First, respondents in this category deflected from racism as a key societal problem by either denying its existence outright or deflecting and distracting from it:

*I do feel that racism isn't dying out, but that it's actually much less prevalent in my generation than it was in my parents‘ generation. And I see that as a positive development. But I also feel that some issues are sometimes given too much attention in the racism debate, which is why they suddenly pop up. I'll try to explain this without sounding negative, but perhaps it's comparable to, yes, when some issues are given too much attention, they may then only arise in the first place*. (0327, female, nurse)

*Since I don't feel that racism is an issue here, I don't have anything that would motivate me to do so. So, no, I can't think of anything that could be done better* (0327, female, nurse)

*We also experience this very, very often, that, um, let's say, the demanding patient, um, who is sometimes told here and there, even here in the emergency room or in the ambulance service, um, that a limit has now been reached between what one can wish for and what we can offer. Um. And then, in turn, the patients like to accuse us of being racists. And I personally (laughs) always find that offensive* (0326, male, attending physician)

The first quote frames racism as a diminishing problem within an increasingly open and purportedly inclusive society. It downplays the continuing significance of racial inequity, positioning discussions about racism as outdated or unnecessarily divisive. Moreover, it delegitimizes those who raise concerns about racism, accusing them of fabricating or amplifying a problem that is perceived to no longer exist. In doing so, this view not only denies the persistence of structural inequities but also shifts the focus from systemic critique to the supposed excesses of those who challenge racial injustice. In the third quote, the respondent links specific challenging behaviors observed in the emergency department to their own racialized assumptions. Difficult behaviors are interpreted as stemming from patients' cultural or ethnic backgrounds, rather than considering other potential contributing factors, such as repeated experiences of racism, chronic stress, or other forms of systemic oppression. The excerpt further reflects a lack of engagement with the respondent's own positionality and whiteness, instead centering the speaker's personal experience of offense. This response exemplifies a defensive posture that prioritizes feelings of being affronted over critical self-reflection, thereby reinforcing biased interpretations of patient behavior.

Orientations of this type also frequently invert perpetrator-victim roles, evident in the last quote above where the focus is quickly shifted onto “the demanding patient” but also evident in other interview excerpts:

*And I believe that many patients come to the hospital with the wrong idea and then have certain expectations or demands that could lead to compartmentalized thinking* (0327, female, nurse)

*The only thing that actually annoys me sometimes, I have to admit, is the language barriers that arise. I find that exhausting and, in some situations, difficult to understand. So, a classic example, a pregnant woman who doesn't speak German comes into the delivery room… and then they're awake and I realize that this woman doesn't understand any of us. I always wonder what motivates people when I know that I will also end up in this hospital situation at some point, not to learn the language beforehand*. (0327, female, nurse)

*What I noticed from time to time in the emergency services is that when you tried to take someone's medical history and they couldn't string a single sentence together in German, … where you sometimes thought, 'Oh, come on, that can't be true'* (0326, male, attending physician)

Here, a dynamic of perpetrator–victim inversion can be observed: the speaker attributes misunderstanding, bias or lack of communication skills due to language barriers to the patients—implicitly positioning them as the source of division or miscommunication. This rhetorical move deflects attention from potential institutional or structural biases within the healthcare setting, reassigning the burden of responsibility to those who may already be disadvantaged or marginalized. Instead of addressing the failure of institutions to provide accessible information or multilingual support, they present those who experience discrimination as the agents of miscommunication rather than its targets.

Accordingly, this type does not regularly engage in reflecting on their privileges vis-à-vis racism:

*No, I don't think I'm more privileged now, and I don't think I'm significantly smarter or anything. No, I don't think I'm more privileged* (0327, female, nurse)

While it is plausible that many respondents lack familiarity with the notion of privilege and, as exemplified by this respondent, misconstrue it as a marker of cognitive or intellectual superiority, their responses reveal a limited reflexivity regarding their own positionality within broader social relations of power and inequality.

In conclusion, the skeptical-dismissive type is the only one characterized by a simultaneous rejection of the existence of structural racism and a consistent minimization of the role of personal responsibility in its reproduction.

Crucially, across the different types discussed here, each profession drew on its own discipline-specific discourses, ways of thinking and core concepts. For example, in medicine, professionals often relied on academic language or the notion of “reflex” (0329) as a biological or clinical concept, implying that the impulse behind racism is inherent and therefore difficult to control. In occupational therapy, by contrast the emphasis was on a reflexive practice, the development of which was built into respondents' training and education and encourages practitioners to critically consider their own actions and assumptions. In a sense though, the presupposition of already-existing reflexivity and the emphasis on client empowerment appeared to constrain respondents from engaging in a more in-depth and critical reflection on their own privilege. In nursing, central ideas such as patient-centered care and a strong professional ethos guided practice, highlighting the moral and relational dimensions of care. This prioritization of individual patient needs and values, emphasizing empathy and shared decision-making, however, could inadvertently lead to the neglect of structural-level drivers such as racism and organizational hierarchies, which shape nursing practice. Together these examples show how disciplinary discourses and practices shape professional identity and practice across fields. At the same time, each profession tended to defend its boundaries and expertise against others, using its distinct language, values and standards to assert legitimacy and protect its professional territory. Such boundary work helps maintain professional identity and authority but can also limit interdisciplinary collaboration.

## 4 Discussion

This article has shown that white German healthcare professionals perceptions of their own conduct toward racialized patients, including the interpretive frameworks and patterns of attribution underlying these perceptions, vary significantly. While some are highly sensitized regarding their privileged social positionality and acknowledge their responsibility in using this privilege to counteract racism in clinical encounters and structures, others deny or deflect from the issue at hand. We have clustered these attitudes and orientations into five different types (critical-reflexive, antiracist, liberal, intentional and skeptical-dismissive). The distinction between critical and antiracist approaches is thereby well-known in the German literature on racism (international scholarship on racism does not appear to make this distinction as clearly): critical approaches seek to continually question the effects of ethno-national-cultural schemes of differentiation, including one's own involvement in them, while antiracist approaches are more oriented toward collective political action [e.g., (40–42)]. The majority of attitudes and orientations thereby fell into the liberal category, reflective of the bodies of knowledge and practices shaped by the policy framework of intercultural dialogue and competency. This framework emphasizes cultural diversity, tolerance, and interpersonal sensitivity, and has long dominated approaches to diversity in the German healthcare system (43). As a result, this type tended to frame racism primarily as a matter of individual attitudes while downplaying or denying its structural and institutional dimensions. Indeed, Iman Attia (44) has usefully distinguished between intercultural and antiracist approaches, highlighting the risk of essentialization in the former. As Hamed et al. (3) contend, such conceptualizations and their associated practices continue to exclude racialized individuals or diminish their entitlement and access to high-quality healthcare. The intentional type acknowledges racism only when it manifests as overt, deliberate acts. While morally condemning explicit discrimination, this type may overlook subtler or systemic forms of racialization, and often fails to reflect on its own positionality or complicity. Both the liberal and intentional approaches, despite differences in framing, illustrate a partial engagement with antiracist practice: they may prompt interventions in response to observable incidents but do not consistently challenge the underlying structures that sustain inequity. Finally, the skeptical-dismissive type closely aligns with what Grada Kilomba has described as the first of five stages of awareness of one's own role in reproducing racism (45): Denial, she writes, is part of different ego defense mechanisms that white people necessarily go through in order to eventually “be able to listen” (45, p. 22) and understand the operation of structural racism.

Despite such differences, what was evident across the different types was respondents' limited vocabulary and specifically insecurity to name discriminatory patterns, especially when they concerned their own behavior or assumptions. Stammering, long pauses or queries to the interviewers about the appropriate terminology or “facticity” of their descriptions reflect not only discomfort but also the lack of public discourse about racism in German healthcare, and Germany more broadly [also (46)]. This combination, discomfort and the absence of a broader discourse around racism, can lead to avoidance rather than engagement and, as a result, reflections on racism frequently remain superficial, centering on overt incidents while overlooking structural and everyday forms of exclusion. It also makes it more difficult to acknowledge personal complicity and to develop a nuanced understanding of racism and how it shapes professional conduct.

These results carry important implications for both future research and practice. First, what the data has shown is that during medical and other health education and training, which encompasses both undergraduate and specialist training, students are exposed not only to the formal curriculum but also to a “hidden curriculum” [(47); also (12, 13, 52)]. This hidden curriculum subtly conveys implicit lessons about professional conduct, social hierarchies, and attitudes toward different patient populations, shaping learners' values and practices beyond what is formally taught. Hegemonic knowledge systems are thus continuously reproduced and implicitly transmitted, reflecting the institutional conditions that sustain them. This raises important questions about the role of medical, nursing and therapeutic training in shaping, reinforcing, or transforming these perspectives. Examining how the types presented in this article may interchange over the course of medical, nursing and therapeutic training constitutes an important avenue for future research in order to reveal how rigid institutional structures influence attitudes toward the possibility of systemic change. What would it mean for the different types if the system supported their processes of self-reflection and provided room for collective learning? A young trainee might enter the field as a critical-reflexive type, aiming to pursue deeper structural interventions. Yet, confronted with persistent structural conservatism, their patience may erode as efforts to effect change meet resistance. This can lead to a gradual transformation into the intentional type—still motivated to create change, but now confined to more visible and limited forms. Could medical training cultivate and sustain students' curiosity by creating spaces for critical reflection and dialogue, and by giving equal priority to internal processes of self-examination as to external professional practice?

Moreover, another commonality amongst the types is the lack of institutional infrastructure to support healthcare professionals' commitment to sustainable change. As such, what becomes evident is that the healthcare system's capacity to bring about deep-rooted structural transformation is limited. As Esther Choo (48) writes, research should move beyond documenting well-known inequities and focus on effective interventions. Racism is embedded throughout healthcare settings, in artwork, textbooks, learning modules, and even equipment. It shapes clinical processes, including race-based algorithms, and is reinforced through decision-making, resource allocation, and social participation. Because it permeates every aspect of organizational life, responses must also be systemic. Structural interventions are therefore essential, as individual efforts alone cannot dismantle entrenched inequities. Possible structural interventions include establishing healthcare centers with an intersectional approach in underserved areas, medical curricula that address structural racism, revisions of biased diagnostic algorithms, and transparent feedback systems to expose implicit bias in clinical practice (48).

Indeed, addressing racism in healthcare requires professionals not only to understand it but also to engage in informed discussions about its effects. One approach to achieving this is the Public Health Critical Race Praxis (PHCRP) (49, 52); also see (54), which provides a structured framework for examining how racism shapes both research and clinical practice. PHCRP emphasizes the need to critically consider racial dynamics at both personal and societal levels and encourages sustained dialogue in professional contexts. This approach would be particularly relevant for what we have described as liberal attitudes and approaches, in which healthcare professionals recognize the relevance of racism but frame it primarily as intolerance or individual prejudice. As a result, efforts may focus on symbolic or performative inclusion rather than redistributing power or reducing racism, and the operation of whiteness as an unmarked advantage frequently remains invisible.

Not least, negotiating interprofessional power asymmetries [e.g., (50)] and deconstructing racist attributions requires sustained practice. As we have argued, meaningful change necessitates structural interventions and policy frameworks targeting the institutional level, enabling all types—and, by extension, all health workers—to contribute effectively to dismantling racism while also providing support for racialized healthcare users and staff. In this way, the article extends existing discussions on both racialized discourse (3) and the self-reflection of health workers (21) by emphasizing the commonalities across types, particularly the shared lack of structural infrastructure necessary for dismantling racism.

### 4.1 Limitations

The findings of this study should be interpreted in light of several limitations. First, the sampling approach likely attracted healthcare professionals already willing to engage with issues of racism and to dedicate time to participate in the interview study. As a result, the perspectives captured here may not reflect the full heterogeneity of attitudes among healthcare professionals in Germany. In addition, other professional groups that often constitute the first point of contact for patients—such as reception or administrative staff—were not included, despite evidence that they also frequently reproduce racism [see (53)]. Similarly, no respondents from the age group 18-29 could be recruited, limiting the extent to which we could compare different attitudes according to age. Moreover, the medium through which interviews were conducted (telephonically, via Webex/Zoom or in-person) varied, with possible effects on social desirability bias; telephonic interviews can be more anonymous, potentially leading participants to provide answers that might otherwise be perceived as less favorable [but see (2)]. However, from the small sample size it is not possible to draw definitive conclusions regarding the presence or extent of social desirability bias. Finally, while our analysis touches on professional self-understandings, future research should more systematically generate what Bohnsack et al. (36) refer to as “sociogenetic types” across occupational groups to deepen insights into profession-specific identities and to support the development of more critical curricula, knowledge frameworks and professional cultures. This includes not only the careful analysis of materials used in the education and training of a range of healthcare professionals, but also the “hidden curriculum” conveyed to each profession. In the same way, an intersectional perspective is essential for a more in-depth analysis of healthcare professionals reflections on racism and responsibility. Integrating this perspective can help uncover the complex positionalities of healthcare workers at multiple axes of privilege and marginalization, thereby providing a more nuanced understanding of how racism operates in practice. Not last, while the broader research project from which this article derives also formulates recommendations to reduce racism in practice—particularly to address the lack of institutional support experienced across all types identified here—a systematic scientific evaluation of existing institutional structures remains necessary to establish trust among affected communities and to ensure that public funds in the healthcare sector are spent effectively.

## 5 Conclusion

This article has examined how healthcare professionals perceive their conduct toward racialized patients, and what interpretive frameworks and patterns of attribution underlie these perceptions. We have demonstrated that even though implicit forms of racism persist, respondents demonstrated varying degrees of reflexivity and awareness regarding the everyday reproduction of racism in clinical encounters, including the recognition of their own roles and responsibilities within these processes. However, even those actively engaged in antiracist struggles often relied on culturalizing discourses and explanatory models, reflecting the persistence of essentialist understandings. While those respondents we have classified as exhibiting a critical-reflexive orientation may offer the greatest potential for advancing antiracist practice, our analysis also highlights how other respondent types may engage in meaningful learning processes that contribute to the reduction of racism. In particular, what is needed is not only greater knowledge about racism but the creation of structural spaces for deeper self-reflection regarding one's own role in its reproduction—especially with respect to the conceptual foundations underlying implicit forms of racism.

## Data Availability

The raw data supporting the conclusions of this article will be made available by the authors, without undue reservation.
